# CD36 plays a critical role in proliferation, migration and tamoxifen-inhibited growth of ER-positive breast cancer cells

**DOI:** 10.1038/s41389-018-0107-x

**Published:** 2018-12-21

**Authors:** Yu Liang, Hao Han, Lipei Liu, Yajun Duan, Xiaoxiao Yang, Chuanrui Ma, Yan Zhu, Jihong Han, Xiaoju Li, Yuanli Chen

**Affiliations:** 1 School of Food and Biological Engineering, Hefei University of Technology, Hefei, China; 2 College of Life Sciences, Key Laboratory of Bioactive Materials of Ministry of Education, State Key Laboratory of Medicinal Chemical Biology, Nankai University, Tianjin, China; 3 First Teaching Hospital of Tianjin University of Traditional Chinese Medicine, Tianjin, China; 4 Tianjin State Key Laboratory of Modern Chinese Medicine, Tianjin University of Traditional Chinese Medicine, Tianjin, China

## Abstract

Tamoxifen inhibits estrogen receptor (ER)-positive breast cancer growth while CD36 potentiates cancer metastasis. The effects of CD36 on proliferation/migration of breast cancer cells and tamoxifen-inhibited ER-positive cell growth are unknown. In this study, we correlated the mortality of breast cancer patients to tumor CD36 expression levels. We also found CD36 was higher in ER-rich (MCF-7>T-47D~ZR-75-30) than ER-negative (MDA-MB-231) cells. CD36 siRNA decreased viability and migration of MCF-7 and MDA-MB-231 cells with more potent effects on MCF-7 cells. Inversely, high expressing CD36 enhanced cell growth/migration. Mechanistically, CD36 increased expression of genes responsible for cell proliferation, migration and anti-apoptosis. CD36 also activated ERα and ER-targeted genes for cell cycles, and phosphorylated ERK1/2 (p-ERK1/2). Tamoxifen inhibited CD36 and p-ERK1/2 in ERα-positive but not ERα-negative cells. Reciprocally, inhibition of MCF-7 cell growth by tamoxifen was attenuated by high expressing CD36. CD36, ERα and p-ERK1/2 expression was higher in tamoxifen-resistant MCF-7 (MCF-7/TAMR) cells than normal MCF-7 cells. However, CD36 siRNA restored the capacity of tamoxifen inhibiting MCF-7/TAMR cell growth. CD36 antibody inhibited cell growth and expression of ERα, p-ERK1/2 and CCND1. Therefore, our study unveils a pro-tumorigenic role of CD36 in breast cancer by enhancing proliferation/migration of breast cancer cells while attenuating tamoxifen-inhibited ER-positive cell growth.

## Introduction

Breast cancer, one of the most common diagnosed cancers, is the second leading cause of cancer death of women in the United States^1^. As a heterogeneous disease, breast cancers can be classified into several subtypes based on their distinct biological, molecular and clinical courses^2,3^. Approximately 75% breast tumors are estrogen receptor-α (ERα) positive, indicating that the prevalence of breast cancers is strongly correlated to ER activation. After binding with the ligand, the activated ER can promote cell proliferation while inhibiting cell apoptosis by regulating expression of the key molecules controlling cell cycles, such as c-myc and cyclin D1^4^.

Tamoxifen functions as a selective estrogen receptor modulator based on the targeted cell types or molecules. As an adjuvant therapy, it has been used for prevention and treatment of patients with breast cancers, particularly with ERα-positive tumors, for several decades^5,6^. Functionally, tamoxifen can inhibit proliferation of ER-positive breast cancer cells by competitively binding to ERα. Tamoxifen also activates apoptosis of breast cancer cells in an ERα-independent manner by regulating several signaling targets including protein kinase C, transforming growth factor β, calmodulin, mitogen-activated protein kinase p38 and c-Jun terminal kinase^7^. Although tamoxifen treatment can substantially reduce the death rate of breast cancer patients^1^, about half of the patients still have poor response to tamoxifen treatment and suffer from the recurrence of tamoxifen-resistant tumors^8^. Thus, identification of the mechanisms responsible for tamoxifen resistance as promising approaches is still important to optimize tamoxifen therapy and improve outcome of the treatment.

CD36 is originally identified as a member of type B scavenger receptor family and an 88-kDa glycosylated membrane protein. It can bind multiple ligands including thrombospondin, fatty acids, anionic phospholipids and oxidized low-density lipoprotein (oxLDL)^9^. The high affinity of CD36 for oxLDL in macrophages implies that CD36 expression can have an important pathophysiological role in formation of macrophage/foam cells and atherosclerosis^10^. Indeed, deficiency of CD36 expression inhibits atherosclerosis in high-fat diet-fed low-density lipoprotein receptor or apolipoprotein E-deficient mice^11,12^.

The recent studies have reported that CD36 expression is also involved in tumorigenesis, but the results are controversial. Clezardin et al.^13^ report that CD36 expression is defective in invasive breast cancers, which suggests that loss of CD36 may facilitate tumor progression and metastasis^13^. In another study, CD36 expression is found decreased by estradiol in hormone-dependent MCF-7 and T-47D breast cancer cell lines^14^. However, more studies have demonstrated the pro-tumorigenic properties of CD36. In glioblastoma, CD36 is highly expressed in the self-renewing tumorigenic cancer stem cells, and activation of CD36 by its ligand, oxidized phospholipids, enhances cell proliferation^15^. In hepatocellular carcinoma (HCC), activation of CD36 expression to enhance the uptake of free fatty acids results in enhanced epithelial–mesenchymal transition and progression of HCC^16^. Recently, CD36 has been found to initiate tumor metastasis under a high nutrient condition in various cancer types, such as oral, breast cancer and melanoma^17^. However, the exact role of CD36 in tumorigenesis, particularly in breast cancer, needs more investigation.

Besides the anti-tumorigenic properties, tamoxifen can have pleiotropic functions including cardioprotection. Our previous report shows that treatment of macrophages with tamoxifen inhibits CD36 expression at the transcriptional level by inactivating peroxisome proliferator-activated receptor-γ (PPARγ). Functionally, tamoxifen inhibits macrophage/foam cell formation and atherosclerosis^10^. In addition, we have reported that although macrophage CD36 expression is activated by progesterone which can partially explain that progesterone attenuates the cardioprotective effects of estrogen in the hormone replacement therapy, CD36 expression in multiple tissues is activated physiologically during the gestation^18^. The studies above also suggest the interaction between CD36 and hormones or hormone receptor modulator. In the present study, we determined if CD36 expression can function on proliferation and migration of breast cancer cells, particularly the ER-positive cells, thereby influencing the effect of tamoxifen on growth of breast cancer cells.

## Results

### CD36 expression enhances breast cancer cell proliferation and migration

**Fig. 1:**
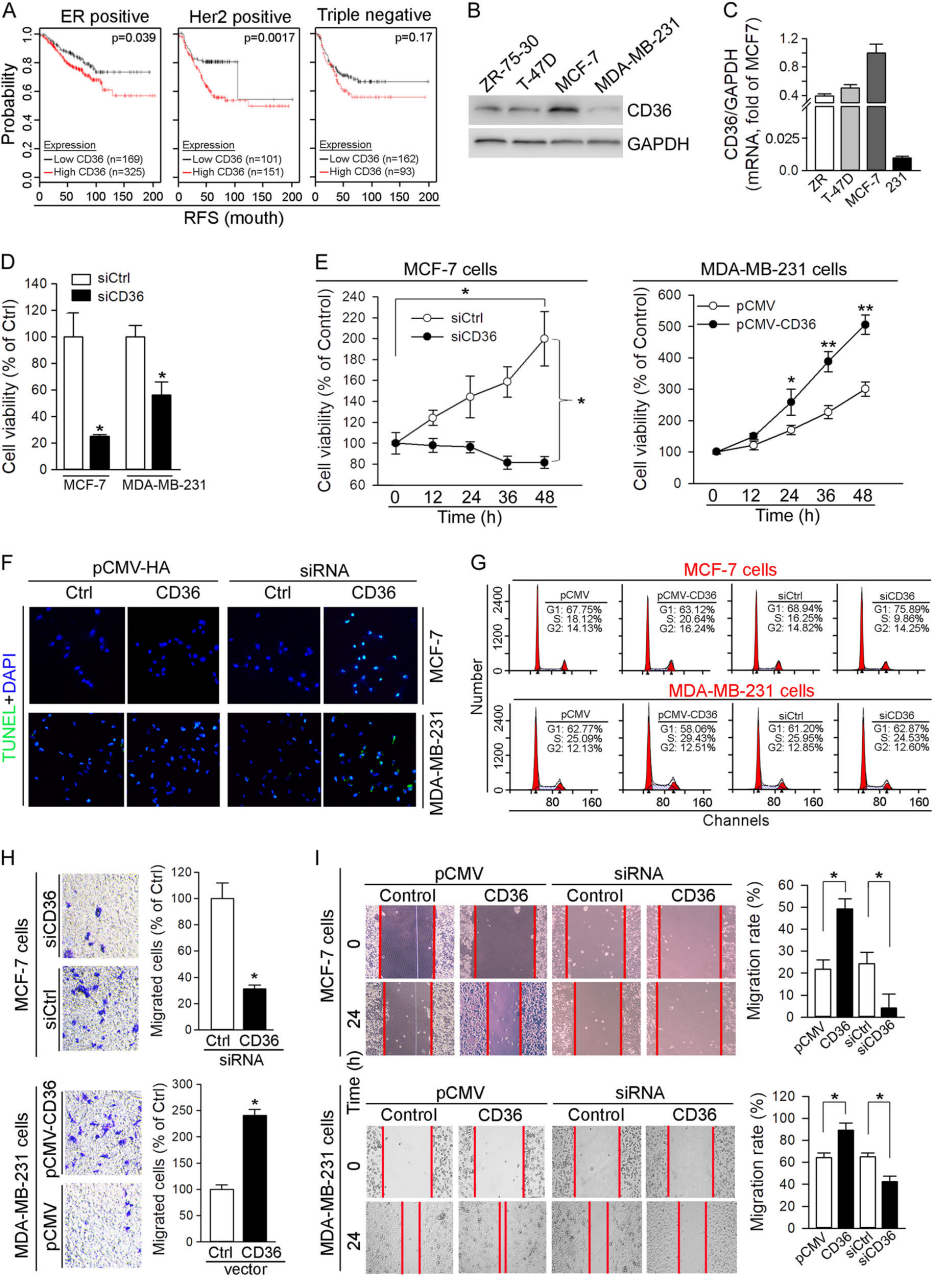
CD36 expression facilitates proliferation and migration of breast cancer cells. **a** CD36 mRNA expression data of ERα-positive (494), Her2-positive (252) or triple-negative (255) breast cancer cases were retrieved from the gene-expression profiling dataset (#206488 from Kaplan–Meier Plot database) and divided into two groups: high CD36 and low CD36 expression in tumors. The data were then used to conduct the Kaplan–Meier analysis, respectively. RFS, the time of recurrence-free survival (month). **b**, **c** Expression of CD36 protein and mRNA in ER-positive (MCF-7, ZR-75-30 and T-47D) and ER-negative (MDA-MB-231) cell lines was determined by western blot (**b**) and qRT-PCR (**c**), respectively. **d** MCF-7 and MDA-MB-231 cells were transfected with control siRNA (siCtrl) or CD36 siRNA (siCD36) for 12 h. Cells were then switched to complete medium and cultured for 72 h, followed by determination of cell viability by MTT assay; *p < 0.05 (*n* = 6). **e**–**g** MCF-7 cells and MDA-MB-231 cells were transfected with siCtrl/siCD36 or pCMV/pCMV-CD36 for 12 or 6 h. After transfection and removal of dead cells, the viable cells were switched into complete medium and cultured for the indicated times, and conducted the determination of cell viability by MTT (**e**), apoptosis by TUNEL (**f**) and cell cycle by FACS assay (**g**); *p < 0.05; **p < 0.01 (*n* = 6). **h** MCF-7 and MDA-MB-231 cells were transfected with siCtrl/siCD36 and pCMV/pCMV-CD36 for 12 and 6 h, respectively. The transfected cells were switched into complete medium and cultured for 24 h. After lifting, ~2 × 10^4^ cells were plated onto the upper compartment of a transwell chamber and continued in culture for 24 h, followed by determination of migrated cells; *p < 0.05 (*n* = 3). **i** MCF-7 and MDA-MB-231 cells in 12-well plates were transfected with pCMV/pCMV-CD36 or siCtrl/siCD36 for 6 or 12 h. Cells were then switched to medium containing 2% FBS and conducted migration assay by the cell scratch test. The photos were taken at the beginning and the end of the scratch test. The width of scratching at 0 h or 24 h was recorded as W_0_ or W_24_. The migration rate was calculated as (W_0_ − W_24_)/W_0_ × 100%; *p < 0.05 (*n* = 5)

CD36 has been demonstrated to be involved in metastasis of various cancer types indicating its pro-tumorigenic properties^17,19^. To determine the correlation between the prognosis of breast cancer and the CD36 expression levels, we retrieved CD36 messenger RNA (mRNA) expression data from the gene-expression profiling dataset (#206488 in Kaplan–Meier Plot database), and completed the Kaplan–Meier analysis on the dataset. As shown in Fig. 1a, the probability with the same value of recurrence-free survival (RFS) was higher in ER- or Her2-positive patients with low tumor CD36 expression than patients having high CD36 expression (*p* = 0.039 or 0.0017). Meanwhile, although the similar trend was observed in the triple negative patients, the difference was not significant (*p* = 0.17) which might be because not enough samples have been collected. Based on the results in Fig. 1a, we believe that CD36 expression can be inversely correlated to the prognosis of breast cancer patients, and CD36 may play an important role in the progression of breast cancers, such as cell proliferation and migration.

To determine if CD36 expression is involved in proliferation or migration of breast cancer cells in an ER-dependent manner and influence the effect of tamoxifen on cell growth, we selected four types of breast cancer cell lines, MCF-7, ZR-75-30, MDA-MB-231 and T-47D cells. Among these cell lines, the growth of MCF-7, ZR-75-30 and T-47D cells can be reduced by tamoxifen, while MDA-MB-231 cells are not sensitive to tamoxifen treatment. We compared CD36 expression in these cell types. As shown in Fig. 1b, c, CD36 expression, either at protein or mRNA level, is highest in MCF-7 cells, while being lowest in MDA-MB-231 cells, indicating the sensitivity of tamoxifen to growth of breast cancer cells is related to cellular CD36 expression. It also implies that CD36 expression can be manipulated as inhibition in MCF-7 cells or activation in MDA-MB-231 cells by transfecting CD36 small interfering RNA (siRNA) or expressing vector. Therefore, we decided to use MCF-7 and MDA-MB-231 cells to complete the rest of experiments in this study.

We transfected both MCF-7 and MDA-MB-231 cells with CD36 siRNA and determined cell proliferation using the 3-(4,5’-dimethylthiazol-2-yl)-2,5’- diphenyltetrazolium bromide (MTT) method. Compared with control siRNA (siCtrl)-transfected cells, inhibition of CD36 expression by siRNA reduced cell growth in both MCF-7 and MDA-MB-231 cells with a greater effect on MCF-7 cells (Fig. 1d). Furthermore, inhibition of CD36 expression by siRNA in MCF-7 cells arrested cell growth and activated cell apoptosis (left panel, Fig. 1e; up panel, Fig. 1f). In contrast, activation of CD36 expression by transfecting CD36 expression vector enhanced the growth of MDA-MB-231 cells (right panel, Fig. 1e). We also determined the cell cycle regulation by CD36 using fluorescence-activated cell sorting (FACS) assay. We found high expressing CD36 reduced the percentage of cells in the G1 phase but increased it in the S phase (left half, Fig. 1g). In contrast, inhibition of CD36 expression by CD36 siRNA increased the percentage of cells in the G1 phage while reducing it in the S phase, particularly in MCF-7 cells (right half, Fig. 1g). These data indicate that CD36 can enhance cell growth by promoting the entrance of cells from G1 phase into S phase.

Next, we determined the effect of CD36 expression on breast cancer cell migration by conducting Transwell and wound healing assays. In MCF-7 cells, high expressing CD36 facilitated while inhibition of CD36 expression almost totally blocked cell migration (up panels of Fig. 1h, i). Similarly, high expressing CD36 enhanced while inhibition of CD36 expression attenuated migration of MDA-MB-231 cells (low panels of Fig. 1h, i). Therefore, the results in Fig. 1 suggest that activation of CD36 expression can promote proliferation and migration of breast cancer cells.

**Fig. 2:**
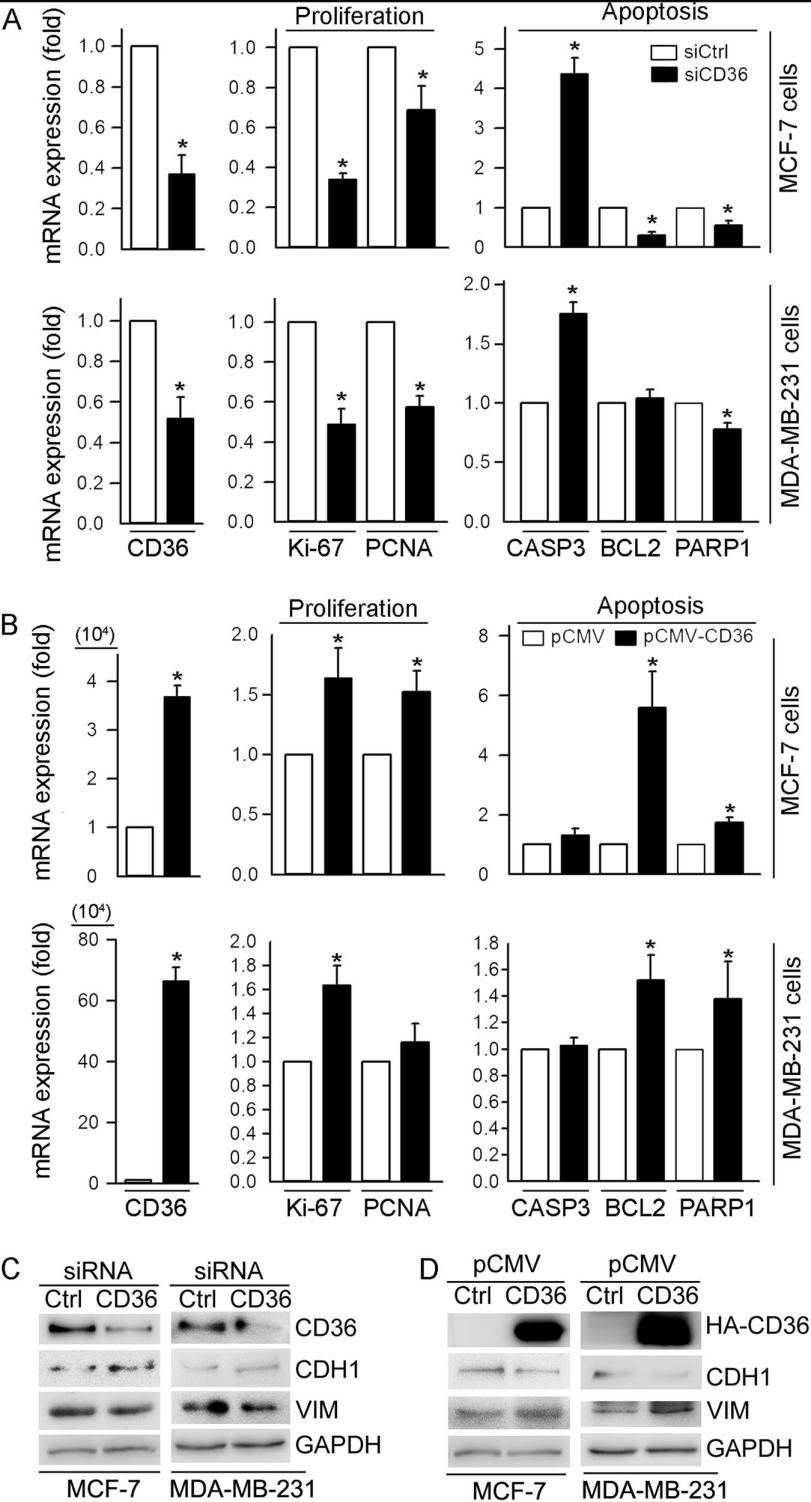
CD36 activates expression of pro-proliferation and migration genes while inhibiting expression of apoptotic genes. MCF-7 and MDA-MB-231 cells were transfected with siCtrl/siCD36 for 12 h (**a**, **c**) or pCMV/pCMV-CD36 for 6 h (**b**, **d**). Cells were then continued culture in complete medium for 48 or 24 h. Total cellular RNA was extracted and used to determine mRNA expression of CD36, the genes related to proliferation (Ki-67, PCNA) and apoptosis (CASP3, BCL2, PARP1) by qRT-PCR (**a**, **b**); *p < 0.05 (*n* = 3). Total protein was extracted and used to determine CD36, HA-CD36, CDH1 and VIM expression by western blot (**c**, **d**)

To disclose the underlying mechanisms by which CD36 enhances cell proliferation/migration, we determined the levels of genes involved in proliferation, migration and apoptosis in response to modulation of CD36 expression in MCF-7 and MDA-MB-231 cells. As shown in Fig. 2a, when CD36 expression was inhibited by CD36 siRNA, expression of Ki-67 and PCNA, two molecules promoting cell proliferation^20^, was reduced in both MCF-7 and MDA-MB-231 cells. In contrast, expression of caspase-3 (CASP3), an enzyme activating cell apoptosis, was increased, while expression of B cell CLL/lymphoma 2 (BCL2) and poly (ADP-ribose) polymerase 1 (PARP1), two anti-apoptotic molecules, was inhibited in MCF-7 cells (up panel, Fig. 2a). Meanwhile, in MDA-MB-231 cells, we determined the similar results except BCL2 which was unaffected by CD36 siRNA (low panel, Fig. 2a).

Reciprocally, high expressing CD36 induced the pro-proliferative phenotype in both MCF-7 and MDA-MB-231 cells. For instance, expression of Ki-67 and PCNA was increased (middle panels, Fig. 2b). In addition, CD36 may protect MCF-7 and MDA-MB-231 cells against apoptosis by increasing BCL2 and PARP1 expression (right panels, Fig. 2b).

Cadherin 1 (CDH1, also known as E-cadherin) and Vimentin (VIM) are genes responsible for cell migration^21,22^. Inhibition of CD36 expression by siRNA increased CDH1 expression but inhibited VIM expression in both MCF-7 and MDA-MB-231 cells (Fig. 2c). Reciprocally, expression of CDH1 or VIM was decreased or increased by high expressing CD36 (Fig. 2d), suggesting enhanced migration of MCF-7 and MDA-MB-231 cells by CD36.

### CD36 activates ERα and ERK1/2 signaling pathways

**Fig. 3:**
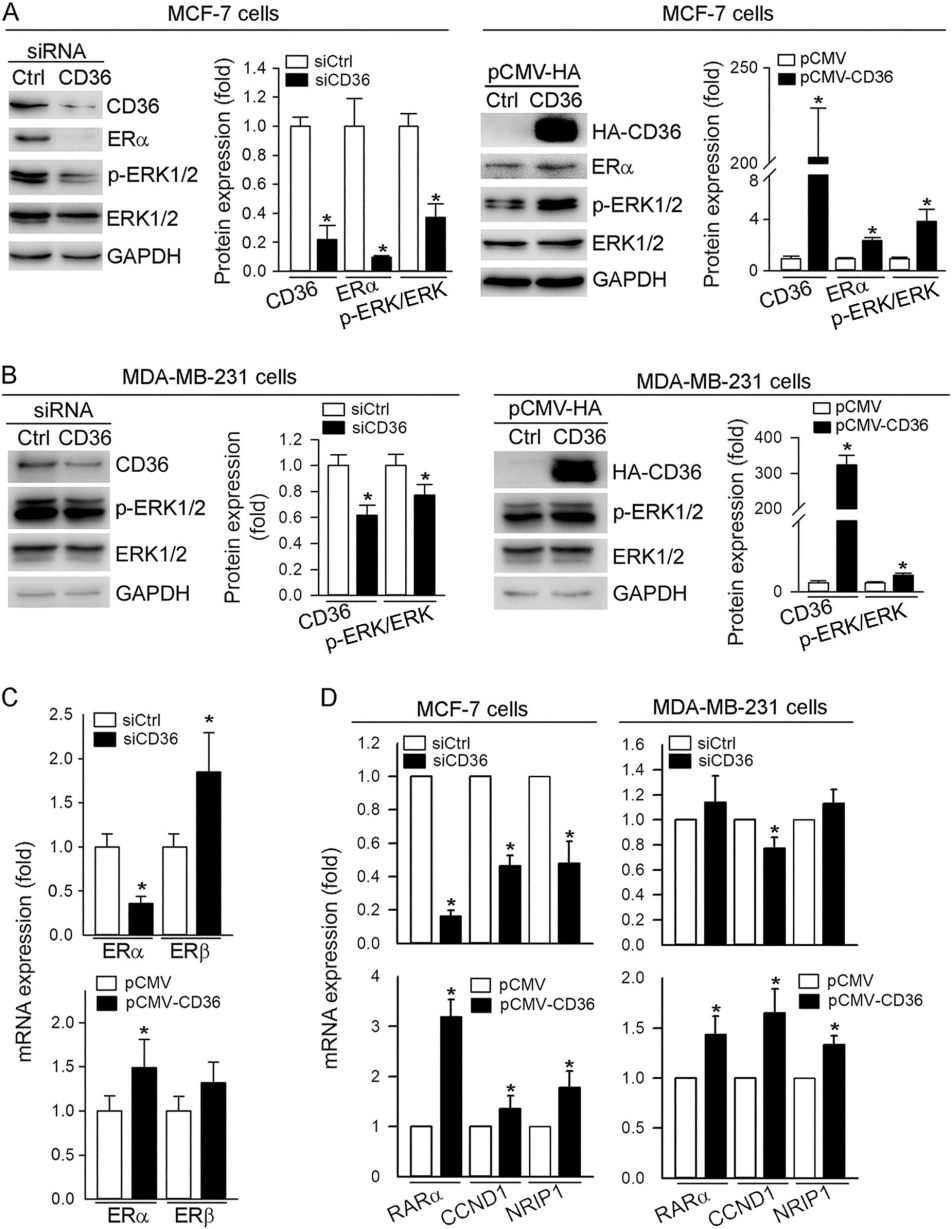
Expression of ERα or ERα-targeted genes and phosphorylation of ERK1/2 are activated by CD36. MCF-7 and MDA-MB-231 cells were transfected with siCtrl/siCD36 for 12 h (left panel) or pCMV/pCMV-CD36 for 6 h (right panel). Cells were then continued in culture in complete medium for 48 or 24 h. Total cellular proteins were extracted to determine expression of CD36, ERα, p-ERK1/2 and ERK1/2 by western blot (**a**, **b**). mRNA expression of ERα/β (**c**) and ERα-targeted genes (RARα, CCND1 and NRIP1) (**d**) in siCtrl/siCD36 or pCMV/pCMV-CD36 transfected cells was determined by qRT-PCR; *p < 0.05 (*n* = 3)

To further define the mechanisms by which CD36 promotes cell proliferation/migration and inhibits apoptosis, we determined if CD36 can activate extracellular signal-regulated kinase-1/2 (ERK1/2; phosphorylated ERK1/2 (p-ERK1/2)) and ERα, the two signaling pathways involved in breast cancer cell growth^23,24^. In MCF-7 cells, reduction of CD36 expression by siRNA inhibited ERα expression which was associated with substantial reduction of p-ERK1/2 and slight reduction of total ERK1/2, which results in significant reduction of the ratio of p-ERK1/2 to ERK1/2 (left panel, Fig. 3a). Meanwhile, high expressing CD36 increased both ERα and p-ERK1/2 (right panel, Fig. 3a). Compared with MCF-7 cells, although CD36 was still able to regulate p-ERK1/2, the effect was much weaker in MDA-MB-231 cells, the cell type lacking ER expression (Fig. 3b). Therefore, regulation of ERK1/2 signaling pathway by CD36 might be completed in both ER-dependent and -independent manners.

Interestingly, in MCF-7 cells, although ERα mRNA expression was activated by CD36, ERβ mRNA expression was not regulated in a similar pattern as ERα. In fact, ERβ mRNA expression was increased by CD36 siRNA but not inhibited by high expressing CD36 (Fig. 3c), which might be because ERβ expression at the basal level in the cells is too low to be further reduced. Associated with changes of ERα or p-ERK1/2, expression of ER-targeted genes, retinoic acid receptor α (RARα), cyclin D1 (CCND1) and nuclear receptor interacting protein 1 (NRIP1) was correspondingly regulated (left panel, Fig. 3d). Meanwhile, the regulation of ER-target genes by CD36 was also seen in MDA-MB-231 cells (right panel, Fig. 3d), indicating CD36 regulated those gene expression by other pathways than ERα. Taken together, Fig. 3 indicates that regulation of breast cancer cell proliferation/migration by CD36 is related to activation of ERK1/2 and ERα signaling pathways.

### Tamoxifen reduces growth of ER-positive breast cancer cells by inhibiting CD36 expression

**Fig. 4:**
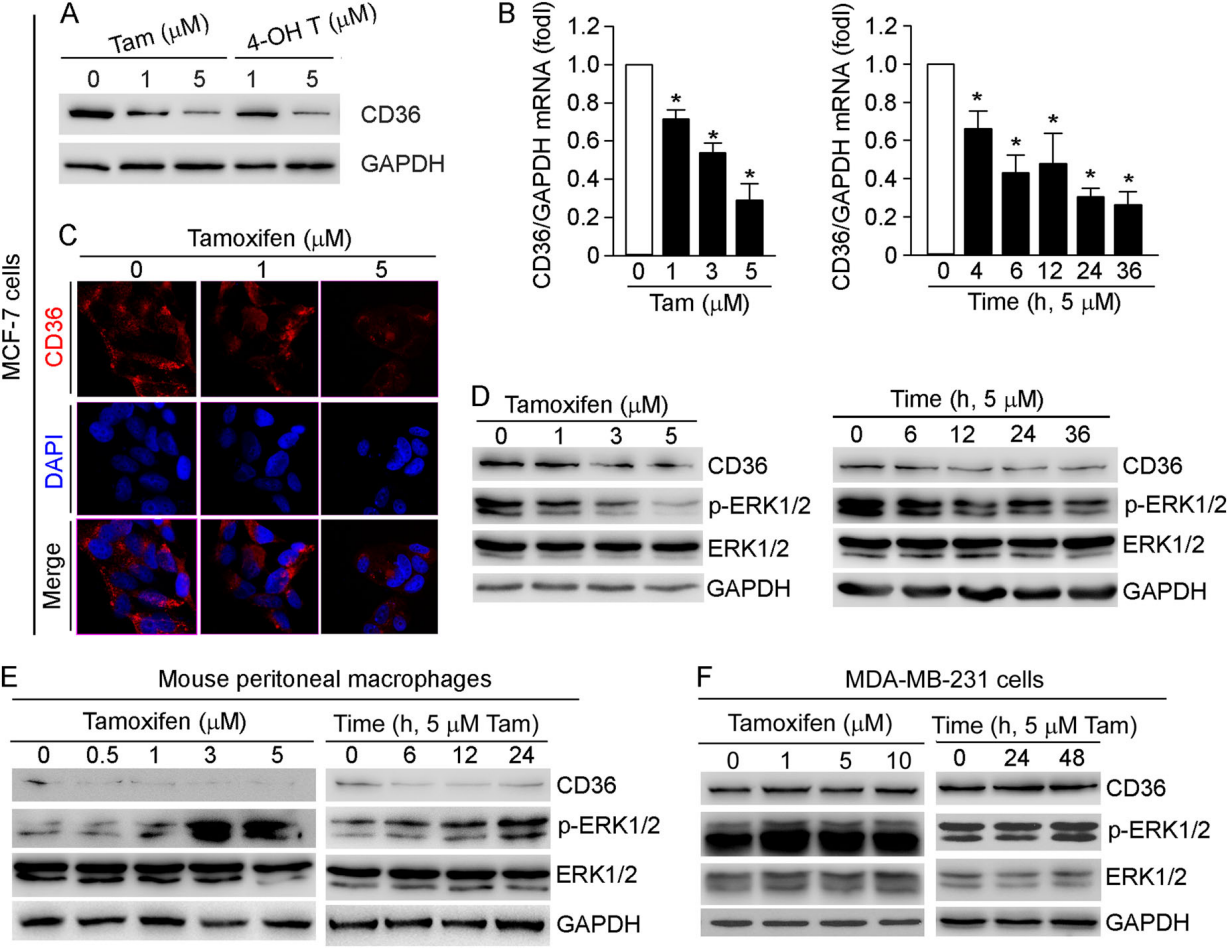
Tamoxifen inhibits expression of CD36 and p-ERK1/2 in ER-positive breast cancer cells. **a**–**d** MCF-7 cells were treated with tamoxifen (Tam) or 4-hydroxytamoxifen (4-OH T) at the indicated concentrations for 16 h, or with 5 μM Tam for the indicated times. Mouse peritoneal macrophages (**e**) and MDA-MB-231 cells (**f**) were treated with tamoxifen at the indicated concentrations for 16 h, or 5 μM Tam for the indicated times. Expression of CD36, p-ERK1/2 and ERK1/2 was determined by western blot (**a**, **d–f**) or immunofluorescent staining (**c**). CD36 mRNA expression was determined by qRT-PCR (**b**); *p < 0.05 (*n* = 3)

Tamoxifen has been used for prevention and treatment of ERα-positive breast cancer in women for more than three decades. It has also demonstrated cardioprotective effects. We previously reported that tamoxifen inhibits macrophage CD36 expression to reduce macrophage/foam cell formation, the initial and critical step in the development of atherosclerosis^10^, which implies that inhibition of CD36 expression may be involved in tamoxifen-inhibited growth of breast cancer cells. In this study, we initially detected that tamoxifen and its active metabolite, 4-hydroxytamoxifen, inhibited CD36 expression in MCF-7 cells in a dose-dependent manner (Fig. 4a). In addition, tamoxifen inhibited CD36 mRNA expression in dose- and time-dependent manners (Fig. 4b). Furthermore, the results of immunofluorescent staining demonstrate cell surface CD36 levels were decreased in response to tamoxifen treatment (Fig. 4c).

Macrophage CD36 expression is regulated by PPARγ, a ligand-activated transcription factor. In addition, phosphorylation of PPARγ by ERK1/2 reduces PPARγ transcriptional activity. Therefore, ERK1/2 inhibitors activate macrophage CD36 expression^25^. Previously, we reported that tamoxifen inhibits macrophage CD36 expression by inactivating PPARγ through activation of ERK1/2^10^. However, in the current study, we found that, associated with inhibition of CD36 expression in MCF-7 cells, tamoxifen reduced p-ERK1/2 in dose- and time-dependent manners (Fig. 4d). In addition, we confirmed that tamoxifen mediated the inverse correlation between CD36 expression and p-ERK1/2 in peritoneal macrophages isolated from male mice (Fig. 4e). Meanwhile, tamoxifen had little effect on either CD36 or p-ERK1/2 in MDA-MB-231 cells (Fig. 4f). Taken together, these data suggest that tamoxifen inhibits CD36 and p-ERK1/2 in a cell type-dependent manner.

**Fig. 5:**
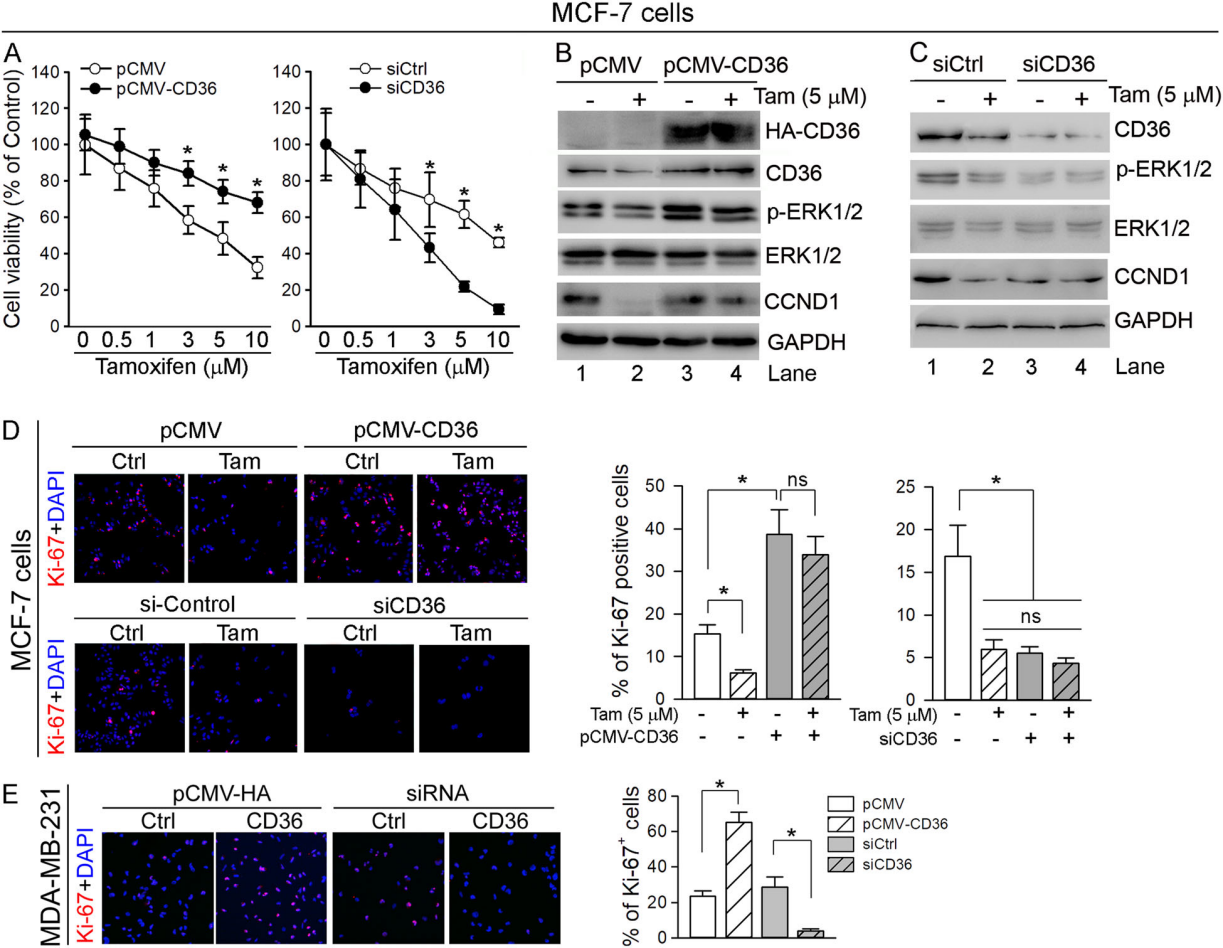
CD36 attenuates tamoxifen-inhibited growth of ER-positive breast cancer cells. **a** MCF-7 cells were transfected with pCMV/pCMV-CD36 or siCtrl/siCD36 for 6 or 12 h, followed by culturing in complete medium for 24 h or 48 h. After removal of dead cells, the viable cells were treated with tamoxifen at the indicated concentrations for 16 h followed by determination of cell viability; *p < 0.05 (*n* = 6). **b** MCF-7 cells were transfected with pCMV or pCMV-CD36 for 6 h, followed by culturing in complete medium for 24 h. Then, the cells was treated with tamoxifen (5 μM) in serum-free medium for 16 h. **c** MCF-7 cells were transfected with siCtrl or siCD36 for 12 h, followed by culture in complete medium for 48 h. Cells were then treated with 5 μM tamoxifen in serum-free medium for 16 h. **d**, **e** MCF-7 and MDA-MB-231 cells in 24-well plates were transfected with pCMV/pCMV-CD36 or siCtrl/siCD36 as described above. MCF-7 cells were also treated with tamoxifen (5 μM) for 16 h. Ki-67 expression was determined by immunofluorescent staining with quantitation of Ki-67-positive cells (>10 fields/sample); *p < 0.05; ns not significantly different (*n* = 10). Expression of CD36, p-ERK1/2, ERK1/2 or CCND1 protein was determined by western blot (**b**, **c**)

To determine if CD36 expression can affect tamoxifen-inhibited ER-positive breast cancer cell proliferation, MCF-7 cells were transfected with CD36 expression vector or CD36 siRNA followed by tamoxifen treatment. Figure 5a shows tamoxifen reduced viability of MCF-7 cells in a concentration-dependent manner, but the reduction was attenuated by high expressing CD36 (left panel). In contrast, inhibition of CD36 expression by siRNA further enhanced tamoxifen-reduced MCF-7 cell viability (right panel), suggesting activation of CD36 expression reduces the inhibitory effect of tamoxifen on growth of MCF-7 cells.

Subsequently, we determined if CD36 expression can influence the effect of tamoxifen on expression of ERα-targeted genes, such as CCND1. In control cells, tamoxifen inhibited CCND1 expression which was associated with reduction of CD36 expression and p-ERK1/2 (Fig. 5b, c; lane 1 vs. 2). However, high expressing CD36 activated p-ERK1/2 while attenuating the inhibitory effect of tamoxifen on CCND1 and p-ERK1/2 (Fig. 5b, lane 3 vs. 1 or 4). Reciprocally, inhibition of CD36 expression by CD36 siRNA reduced p-ERK1/2 and CCND1 (Fig. 5c, lane 3 vs. 1) and blocked the effect of tamoxifen on expression of these genes (Fig. 5c, lane 3 vs. 4). In addition, the results of immunofluorescent staining (Fig. 5d) demonstrate that tamoxifen reduced the number of Ki-67^+^ cells, which was increased by high expressing CD36. In contrast, reduction of CD36 expression by siRNA decreased Ki-67^+^ cells. In addition, either high expressing CD36 or CD36 siRNA disabled the effect of tamoxifen on Ki-67^+^ cells since the cellular CD36 expression cannot be regulated in the transfected MCF-7 cells. Similarly, high expressing CD36 increased while CD36 siRNA decreased Ki-67-positive cell number in MDA-MB-231 cells (Fig. 5e).

**Fig. 6:**
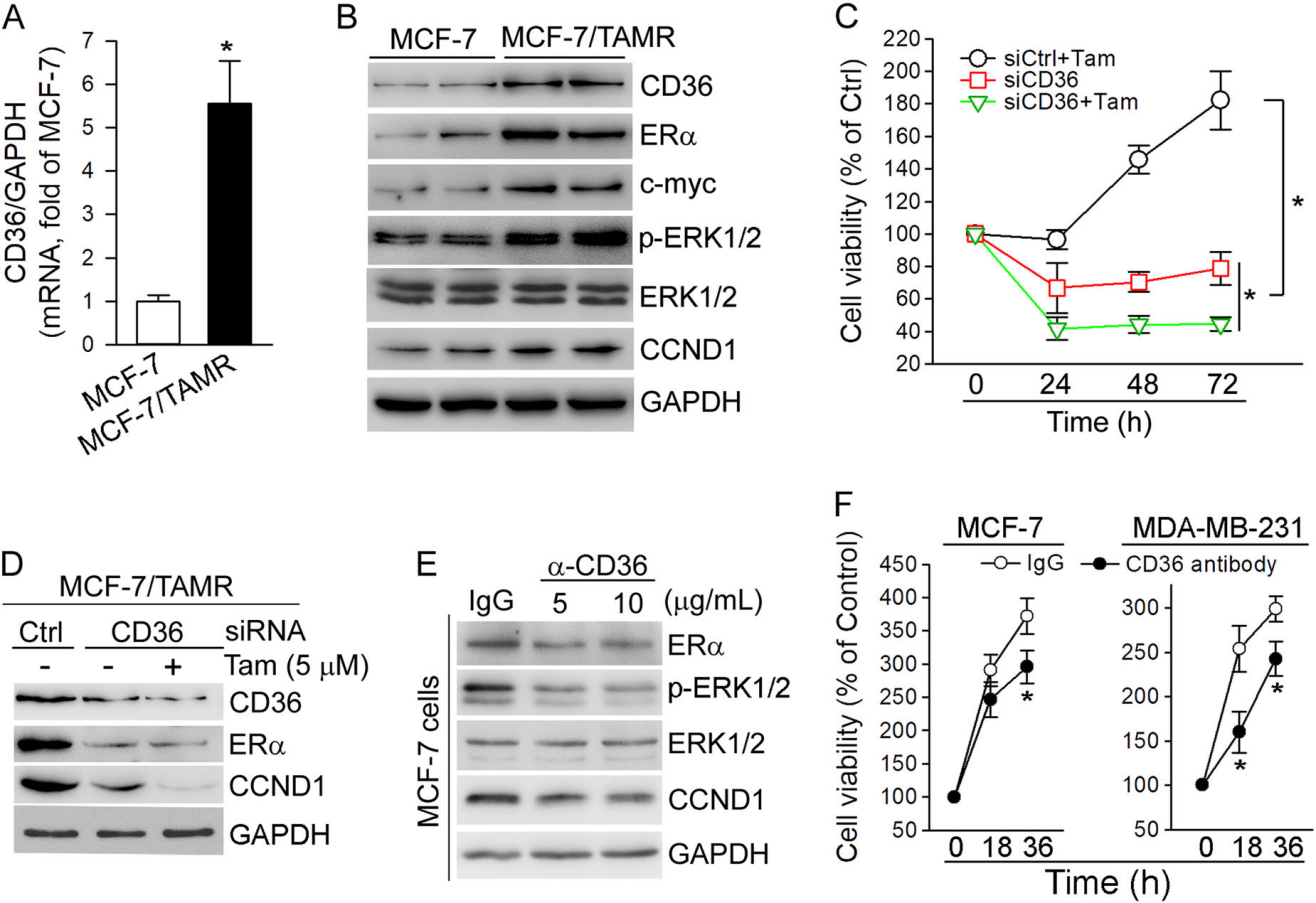
Inhibition of CD36 expression restores the capacity of tamoxifen inhibiting growth of MCF-7/TAMR cells. **a**, **b** Total RNA and proteins were extracted from MCF-7 and MCF-7/TAMR cells. **c** MCF-7/TAMR cells were transfected with siCtrl or siCD36 for 12 h. After removal of dead cells, the viable siCD36 transfected were treated with tamoxifen (5 μM) for the indicated times with determination of cell viability; *p < 0.05 (*n* = 6). **d** MCF-7/TAMR cells were transfected with siCtrl or siCD36 for 12 h, followed by culture in complete medium for 48 h, and then the cells received tamoxifen (5 μM) treatment for 24 h. **e**: MCF-7 cells was treated with rabbit normal IgG or anti-CD36 antibody at the indicated concentrations for 24 h. **f** MCF-7 and MDA-MB-231 cells were treated with rabbit normal IgG or anti-CD36 antibody (10 μg/ml) for 24 h. Expression of CD36, p-ERK1/2, ERK1/2, CCND1, ERα or c-myc protein was determined by western blot (**b**, **d**, **e**); CD36 mRNA expression was determined by qRT-PCR (**a**); *p < 0.05 (*n* = 3). Cell viability was determined by MTT assay (**f**); *p < 0.05 (*n* = 6)

To further correlate the sensitivity of tamoxifen on cell growth with CD36 expression, we initially compared CD36 mRNA and protein expression between normal MCF-7 cells and a tamoxifen-resistant MCF-7 (MCF-7/TAMR) cell line. CD36 mRNA or protein expression was substantially higher in MCF-7/TAMR cells than MCF-7 cells (Fig. 6a, b), which was associated with increased ERα, c-myc, p-ERK1/2 and CCND1 (Fig. 6b). Next, we transfected MCF-7/TAMR cells with CD36 siRNA and determined cell growth in response to tamoxifen treatment. As shown in Fig. 6c, MCF-7/TAMR cells kept growing in the presence of tamoxifen. Inhibition of CD36 expression by siRNA reduced cell number while the inhibition regained the capacity of tamoxifen inhibiting cell growth. Accordingly, we determined that CD36 siRNA inhibited CCND1 expression, which was further enhanced by tamoxifen (Fig. 6d).

Besides siRNA, we determined that neutralization of cellular CD36 function by antibody can reduce p-ERK1/2 and CCND1 expression (Fig. 6e). Functionally, CD36 antibody reduced growth of MCF-7 and MDA-MB-231 cells (Fig. 6f).

## Discussion

Activation of CD36 expression may enhance progression and metastasis of several types of cancers including breast cancer. Based on the correlation between the prognosis of breast cancer and CD36 expression in the tumor (Fig. 1a), we determined that high expressing CD36 increased growth and migration of breast cancer cells (Fig. 1e, h, i), while inhibition of CD36 expression resulted in reduction of growth or migration and increase of apoptosis, mainly in ER-positive breast cancer cells (Fig. 1d–i). We also characterized the effect of CD36 on expression of genes for proliferation, migration and apoptosis in breast cancer cells (Fig. 2). Mechanistically, we observed that pro-proliferative effects of CD36 is completed by activating ERK1/2 and ERα (Fig. 3). Interestingly, we determined that reduction of CD36 expression by siRNA inhibited ERα expression while inducing ERβ expression (up panel, Fig. 3c), which is consistent with the observations that ERα promotes while ERβ inhibits breast cancer cell growth, and the concept that increased ERα and decreased ERβ might be one of the reasons for initiation of breast cancer. It also indicates that CD36-induced ERβ expression may make contribution to the inhibitory effect of siCD36 on cell proliferation.

Tamoxifen can suppress breast cancer cell proliferation. Previously, we reported that tamoxifen reduces CD36 expression in other cell types^10^. In this study, we observed that similar to macrophages, tamoxifen reduced CD36 expression in MCF-7 cells, but not in MDA-MB-231 cells (Fig. 4). Associated with inhibition of CD36 expression, tamoxifen inactivated ERK1/2 in MCF-7 cells while activating it in macrophages and having no effect in MDA-MB-231 cells (Fig. 4d–f). Therefore, tamoxifen regulates CD36 expression in cell type-dependent manner by different signaling pathways. More importantly, we identified that CD36 expression played a critical role determining the sensitivity of tamoxifen on growth of breast cancer cells. Indeed, high expressing CD36 attenuated inhibition of breast cancer cell growth by tamoxifen, while inhibition of CD36 synergized tamoxifen-induced cell death (Fig. 5a, d), which is associated with regulation of ERK1/2 activity correspondingly (Fig. 5b, c). In addition, we demonstrated that increased resistance of MCF-7 cells to tamoxifen is associated with increased CD36 expression, and specific inhibition of CD36 expression by siRNA not only reduced cell growth, but also restored the inhibitory effect of tamoxifen on cell growth (Fig. 6a–d). In addition, dysfunction of CD36 by antibody also reduces cell growth which is associated with inactivation of ERK1/2 (Fig. 6e). Therefore, our study unveils an important role of CD36 in regulation of growth and migration of breast cancer cells and the underlying mechanisms. Our study also suggests an inverse correlation between CD36 expression and sensitivity of tamoxifen on growth of breast cancer cells.

Similar to other types of cancers, the stronger metabolic status exists in breast cancer than normal tissues with an unusual high requirement for fatty acids. Besides as components of cellular membrane, fatty acids can sustain cell dividing and proliferation, fulfill the energy requirement and provide metabolites for anabolic processes. Many studies have defined the importance of enhanced de novo fatty acid synthesis and exogenous supplement of fatty acids for breast cancer cell proliferation. For instance, stearoyl-CoA desaturase 1 (SCD1) catalyzes production of monounsaturated fatty acids from saturated fatty acids. SCD1 has been recognized as a critical target in lung cancer tumor-initiating cells^26^. In MCF-7 cells, inhibition of SCD1 also blocks cell growth and migration, while supplement of exogenous oleic acid reverses cell growth/migration^27^. Clinically, obesity is considered as a risk factor for development of breast cancer and correlates to the poor prognosis^28,29^. Several molecular mechanisms, such as increased levels of estrogens by adipose tissue, inflammatory cytokines, insulin resistance, adipokines and oxidative stress, are involved in obesity-related pathogenesis of breast cancer^30^.

CD36 can bind and internalize fatty acids, particularly the long-chain fatty acids. The following intracellular fatty acid trafficking can be completed by fatty acid binding proteins (FABPs). CD36 is highly expressed in human breast cancer samples. Activation of breast cancer cell growth by oleic acid is correlated to its cellular uptake through CD36 action^27^. Activation of MCF-7 cell proliferation by exogenous FABP4 is associated with increased CD36 expression^31^. Enhancement of CD36 expression or function is also involved in progression of other cancer types. Fatty acid-enhanced epithelial–mesenchymal transition in hepatocellular carcinoma is mediated by CD36 function^16^. Induction of adipocyte CD36 expression results in enhanced ovarian cancer progression and metastasis and drives glioblastoma progression^15,19^.

Activation of ERK1/2 may play an important role in proliferation or apoptosis of MCF-7 cells. For instance, induction of MCF-7 cell proliferation and invasion by apelin-13 is mediated through activation of ERK1/2^32^. Induction of MCF-7 cell apoptosis by tamoxifen is related to inhibition of ERK1/2^33^. In this study, we determined that regulation of ERK1/2 activity is important for CD36-mediated growth of breast cancer cells. We found that inhibition of CD36 expression by siRNA reduced p-ERK1/2 while high expressing CD36 increased it (Fig. 3a, b). In addition, our results show that inhibition of p-ERK1/2 by tamoxifen is ERα dependent (Fig. 4d–f). Tamoxifen inhibited growth of MCF-7 cells and the inhibition was either attenuated by high expressing CD36 or enhanced by CD36 siRNA (Fig. 5a, d). The inhibition of MCF-7 cell growth by tamoxifen is also associated with reduction of CD36 and p-ERK1/2 (Fig. 4a–d). In contrast to MCF-7 cells, tamoxifen had little effect on both CD36 and p-ERK1/2 in MDA-MB-231 cells (Fig. 4f). Although tamoxifen reduced CD36 expression in peritoneal macrophages isolated from male mice, it increased p-ERK1/2 (Fig. 4e). In addition, higher expression of CD36, ERα and p-ERK1/2 was observed in MCF-7/TAMR cells than normal MCF-7 cells (Fig. 6a, b).

As an endocrine therapy, tamoxifen has been applied to ERα-positive breast cancer patients for many years. However, a certain number of patients can eventually develop tamoxifen resistance. In this study, we demonstrated that the effect of tamoxifen on growth of ERα-positive MCF-7 cells is linked to regulation of CD36 expression. Activation of CD36 expression reduced the inhibitory effect of tamoxifen on cell growth, while inhibition of CD36 by siRNA resulted in synergistic inhibition on cell growth. The role of CD36 in cell growth or resistance to tamoxifen treatment is correlated with CD36-mediated uptake of fatty acids to satisfy the activated metabolic status of tumor cells. Inhibition of CD36 expression by siRNA or dysfunction of CD36 by antibody reduced breast cancer cell growth, which suggests that the modulation of CD36 expression might be a potential therapeutic approach to enhance tamoxifen therapeutic effect on tumors.

## Materials and methods

### Materials

Rabbit anti-CD36 (Cat. No.: 18836-1-AP) and glyceraldehyde 3-phosphate dehydrogenase (GAPDH; Cat. No.: 10494-1-AP) polyclonal antibodies were purchased from Proteintech Group, Inc. (Rosemont, IL, USA). Rabbit anti-ERα (Cat. No.: sc-7207) polyclonal antibody was purchased from Santa Cruz Biotechnology (Dallas, TX, USA). Rabbit anti-ERK1/2 (Cat. No.: 9102S) and p-ERK1/2 (Cat. No.: 9101S) polyclonal antibodies and mouse anti-CDH1 (Cat. No.: 14472) and rabbit anti-VIM (Cat. No.: 5741) monoclonal antibodies were purchased from Cell Signaling Technology (Beverly, MA, USA). Rabbit anti-Ki-67 (Cat. No.: ab16667) polyclonal antibody was purchased from Abcam (Cambridge, MA, USA). Mouse anti-HA (Cat. No.: KM8004) polyclonal antibody was purchased from Sungene Biotech Co., Ltd (Tianjin, China). Rabbit antic-myc (Cat. No.: A1309) and CCND1 (Cat. No.: A11022) polyclonal antibodies were purchased from ABClonal Technology (Wuhan, China). Tamoxifen, 4-hydroxytamoxifen and other chemicals were purchased from Sigma-Aldrich (St. Louis, MO, USA).

### Cell culture

MCF-7, ZR-75-30, T-47D (ER-rich) and MDA-MB-231 (triple (ER, PR and HER2) negative) breast cancer cell lines were purchased from ATCC (Manassas, VA, USA). MCF-7 and ZR-75-30 cells were cultured in Dulbecco's modified Eagle's medium (DMEM), while MDA-MB-231 and T-47D cells were cultured in RPMI-1640 medium containing 10% fetal bovine serum (FBS) and 50 μg/ml penicillin/streptomycin, respectively. Tamoxifen-resistant MCF-7 (MCF-7/TAMR) cell line was kindly provided by Professor Tao Zhu from the University of Science and Technology of China (Hefei, China), and cultured in complete RPMI-1640 medium. Cells at ~80% confluences received indicated treatment.

Peritoneal macrophages were collected from male C57BL/6J mice as previously described^18^. After confirming by immunofluorescent staining with anti-CD68 (the marker for macrophages) antibody, macrophages were cultured in complete RPMI-1640 medium for 2 days before treatment.

### Preparation of CD36 expression vector or siRNA and transfection

The complementary DNA (cDNA) encoding human CD36 was generated by reverse transcription (RT) followed by PCR using total RNA extracted from MCF-7 cells and the following primers: forward, 5′-AAATCTAGAGATGGGCTGTGACCGGAACTGTGG-3′; backward: 5′-CCACTCGAGTTATTTTATTGTTTTCGATCTGC-3′. The RT-PCR product was digested with *Xba*I and *Xho*I, and then subcloned into an expression vector pCMV. The plasmid was named as pCMV-CD36 after the sequence and protein expression were confirmed. Cells at ~60% confluence in 96- or 12-well plates were transfected with pCMV empty vector or pCMV-CD36 using Lipofectamine 2000 (Thermo Fisher Scientific Inc., Carlsbad, CA, USA) for 6 h, then received the indicated treatment.

CD36 siRNA was prepared as follows: 3 siRNAs for the different targeting sites in human CD36 mRNA were selected and synthesized with the *Silencer*^*®*^ siRNA construction kit purchased from Thermo Fisher Scientific Inc. and the following templates: site #1, 5′-AAACTTCTGAACATGTTTGCCCCTGTCTC-3′; site #2, 5′-AACACAGGGATTCCTTTCAGACCTGTCTC-3′; and site #3, 5′-AAATATGGCCTAATATGTAACCCTGTCTC-3′. After synthesis, these 3 siRNAs were mixed at 1:1:1 to compose a CD36 siRNA mix which was named as siCD36. The siCtrl was generated using the following template: 5’-AAACCGGTTGATTAGACTCTCCCTGTCTC-3’. Cells at ~60% confluence were transfected with siCtrl or siCD36 for 12 h using Lipofectamine RNA iMAX (Thermo Fisher Scientific Inc.) followed by indicated treatment.

### Detection of cell viability by MTT assay

After indicated transfection or plus treatment, cells in each well (96-well plates) were added with 40 μl MTT solution (5 mg/ml) and incubated for 4 h. The purple formazan crystals formed were dissolved by adding dimethyl sulfoxide (150 μl/well) followed by read of absorbance at 570 nm.

### Determination of protein or mRNA expression by western blot or quantitative real-time PCR (qRT-PCR)

After indicated treatment, cells were washed with phosphate-buffered saline (PBS) and then lysed with a protein lysis buffer to extract total cellular proteins^34^. After determination of concentrations, the same amount of total proteins from each sample was used to determine expression of CD36, hemagglutinin (HA)-labeled CD36 (HA-CD36), p-ERK1/2, ERK1/2, ERα, CDH1, VIM, BCL2, c-myc or CCND1 by western blot^34^.

To determine mRNA expression, after indicated treatment, total RNA was extracted using the Trizol reagent (Life Technologies, Carlsbad, CA, USA)^34^. After synthesis of cDNA using the reverse transcription kit (New England Biolabs, Ipswich, MA, USA), mRNA expression was determined by qRT-PCR with the SYBR green PCR master mix (Bio-Rad, Los Angeles, CA, USA) and primers with the sequences listed in Table 1, and normalized to GAPDH mRNA in the corresponding samples.

**Table 1:** Sequences of primers for real-time RT-PCR

Gene	Sense	Anti-sense
*BCL2*	5′-GATAACGGAGGCTGGGATGC-3′	5′-TCACTTGTGGCCCAGATAGG-3′
*CASP3*	5′-GCTCATACCTGTGGCTGTGTA-3′	5′-ACCTTTATTAACGAAAACCAGAGCG-3′
*CCND1*	5′-TATTGCGCTGCTACCGTTGA-3′	5′-CCAATAGCAGCAAACAATGTGAAA-3′
*CD36*	5′-TCACTGCGACATGATTAATGGTAC-3′	5′-ACGTCGGATTCAAATACAGCATAGAT-3′
*ERα*	5′-TATGTGTCCAGCCACCAACC-3′	5′-GGTCTTTTCGTATCCCACCTT-3′
*ERβ*	5′-GCCGACAAGGAGTTGGTACA-3′	5′-GGTCAATTGAGCGCCACATC-3′
*GAPDH*	5′-TGATGACATCAAGAAGGTGGTGAAG-3′	5′-TCCTTGGAGGCCATGTGGGCCAT-3′
*Ki-67*	5′-TGACTTCCTTCCATTCTGAAGAC-3′	5′-TGGGTCTGTTATTGATGAGCC-3′
*NRIP1*	5′-GCTGGGCATAATGAAGAGGA-3′	5′-CAAAGAGGCCAGTAATGTGCTATC-3′
*PARP1*	5′-CACCCAGAGTCTTCTCTGCC-3′	5′-GAGGTGGATGGGTTCTCTGA-3′
*PCNA*	5′-AAGAGAGTGGAGTGGCTTTTG-3′	5′-TGTCGATAAAGAGGAGGAAGC-3′
*RARA*	5′-CCTGAATCGAGCTGAGAGGG-3′	5′-TGTGATGCTGCTCAGGTGTG-3′

### Determination of CD36 and Ki-67 expression by immunofluorescent staining

Cells (~80% confluence) cultured on cover slips in 24-well plates received indicated treatment. After removal of medium, cells were fixed in 4% paraformaldehyde for 30 min before conduction of immunofluorescent staining with anti-CD36 or Ki-67 antibody^34^.

### Determination of cell migration by cell scratch test and Transwell assay

MCF-7 or MDA-MB-231 cells (~90% confluence) in 12-well plates received indicated transfection for 6 h. Cells were then wounded using a plastic tip and washed twice with PBS to remove the suspended cells or cell debris. After adding with medium containing 2% FBS, cells were photographed under a light microscope (Leica, Wetzlar, Germany) and the width of scratching was recorded as W_0_. Cells were continued in culture for another 24 h followed by photograph and recording the width of scratching uncovered as W_24_. The migration rate was calculated as (W_0_ − W_24_)/W_0_ × 100%.

Cell migration was also determined by the method of Transwell assay using the transwell chambers purchased from BD Biosciences (Franklin Lakes, NJ, USA). After indicated transfection, cells were lifted and resuspended in DMEM containing 2% FBS. About 2 × 10^4^ cells were transferred into the upper compartment of each well in the chamber. Meanwhile, the lower compartment of each well was added with 0.5 ml complete (10% FBS) DMEM. After 24 h of incubation and removal of the non-invasive cells, the upper compartment was fixed and stained with 0.1% crystal violet for 30 min. After being photographed, the crystal violet was extracted by adding 33% acetic acid solution and absorbance of the extracted solution at 570 nm was read.

### Kaplan–Meier survival analysis

CD36 mRNA expression values were retrieved from an ER-positive, Her2-positive or triple-negative breast cancer profiling metaset deposited in the Kaplan–Meier Plot (#206488). The cumulative survival probability was evaluated using the Kaplan–Meier method^17^.

### Determination of apoptosis by TUNEL assay

MCF-7 and MDA-MB-231 cells were transfected with pCMV-CD36 or siCD36 as described above. Then, cell apoptosis was determined by using a TUNEL (terminal deoxynucleotidyl transferase dUTP nick end labeling) assay kit based on the instruction from the manufacturer (Vazyme Biotech Co.,Ltd, Nanjing, China, Cat. No.: A111-02). All images of the cells were obtained with a fluorescence microscope (Leica DM5000B).

### Determination of cell cycle by FACS assay

After the indicated treatment, about 3 × 10^6^ cells were collected and fixed in 70% ethanol for ~24 h. After washing twice with PBS, cells were added with RNase (1 mg/ml) and incubated for 30 min at 37 °C. Cells were then stained with propidium iodide solution (2 mg/ml) for 30 min, followed by determination of cell cycle by FACS assay.

### Statistical analysis

All data were generated from at least three independent experiments. The quantification of all western blot band density and Ki-67 immunofluorescent staining was analyzed by two individuals who are blinded to the experimental design and each other’s results. The data in normal distribution, which were determined by the one-sample Kolmogorov–Smirnov of non-parametric test with SPSS 22 software (IBM), were analyzed by a parametric statistic (two-tailed Student's *t*-test for two groups and one-way analysis of variance for more than two groups). Data were presented as means ± SEM with significant difference at *p* < 0.05.
